# Crystal structure of (4-hy­droxy­piperidin-1-yl)(4-methyl­phen­yl)methanone

**DOI:** 10.1107/S2056989015018307

**Published:** 2015-10-03

**Authors:** B. K. Revathi, D. Reuben Jonathan, K. Kalai Sevi, K. Dhanalakshmi, G. Usha

**Affiliations:** a PG and Research Department of Physics, Queen Mary’s College, Chennai-4, Tamilnadu, India; b Department of Chemistry, Madras Christian College, Chennai-59, India; c SCRI, Anna Hospital Campus, Chennai-106, Tamilnadu, India; d Anna Siddha Medical College, Chennai-106, Tamilnadu, India

**Keywords:** crystal structure, piperdine derivative, hydrogen bomding

## Abstract

In the title compound, C_13_H_17_NO_2_, the dihedral angle between the planes of the piperidine and benzene rings is 51.7 (2)°. The bond-angle sum around the N atom [359.8 (3)°] indicates *sp* ^2^ hybridization of the atom. In the crystal, O—H⋯O hydrogen bonds link the mol­ecules, forming chains along [001].

## Related literature   

For the biological activity of piperdine derivatives, see: Pissamitski *et al.* (2007 ▸); Katritzky *et al.* (1995 ▸); Dimmock *et al.* (2001 ▸); Watson *et al.* (2000 ▸); Thomas *et al.* (1998 ▸); Sambath *et al.* (2004 ▸). For related structures, see: Revathi *et al.* (2015 ▸); Prathebha *et al.* (2015 ▸). For the synthesis, see: Revathi *et al.* (2015 ▸).
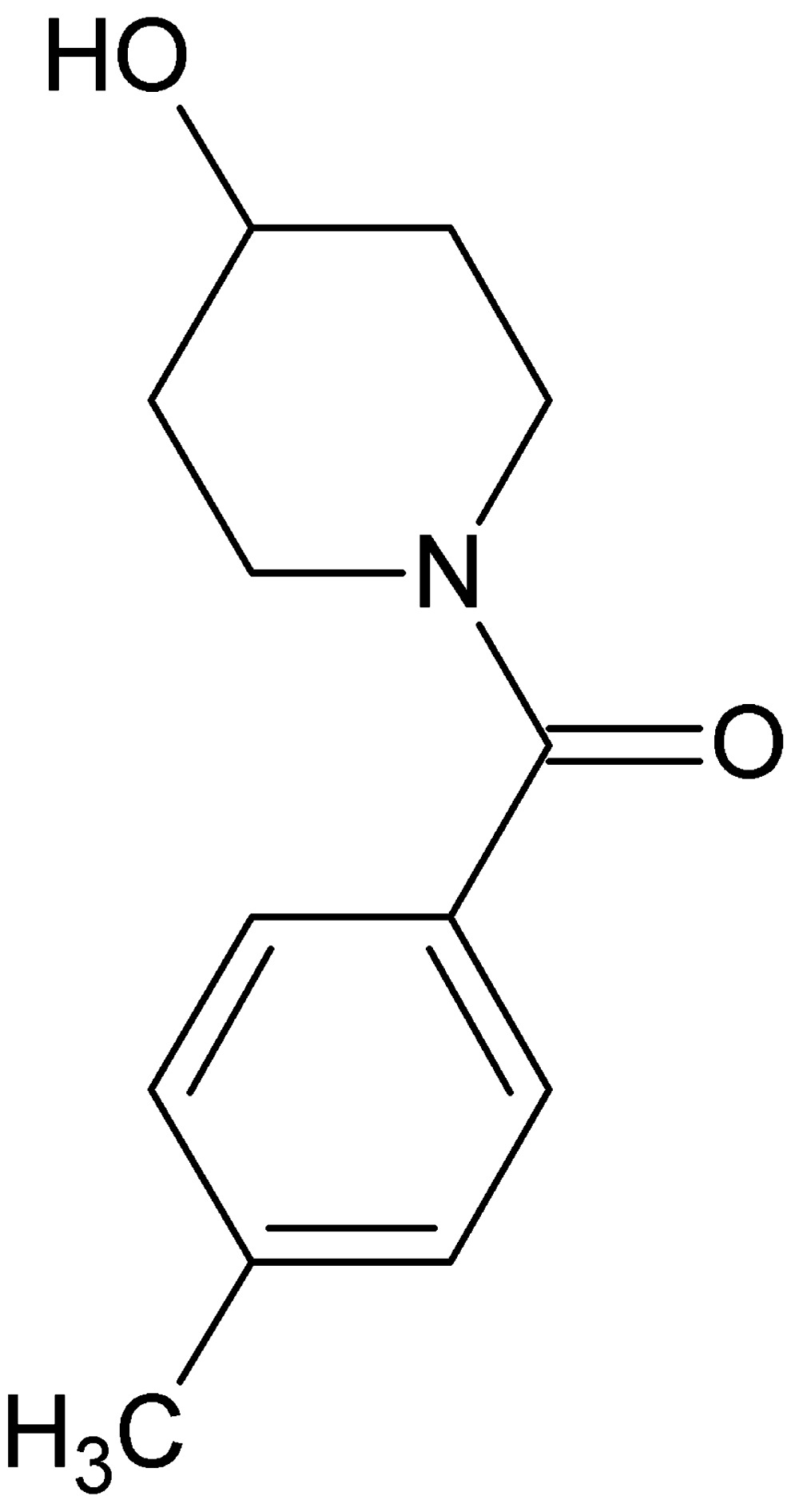


## Experimental   

### Crystal data  

C_13_H_17_NO_2_
*M* *_r_* = 219.28
Orthorhombic, 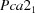

*a* = 23.933 (5) Å
*b* = 6.3317 (12) Å
*c* = 8.0269 (14) Å
*V* = 1216.3 (4) Å^3^
*Z* = 4
Mo *K*α radiation
μ = 0.08 mm^−1^
*T* = 293 K
0.24 × 0.22 × 0.22 mm

### Data collection  

Bruker Kappa APEXII CCD diffractometer
Absorption correction: multi-scan (*SADABS*; Bruker, 2004 ▸) *T*_min_ = 0.981, *T*_max_ = 0.985
10595 measured reflections
3454 independent reflections
1668 reflections with *I* > 2σ(*I*)
*R*_int_ = 0.039

### Refinement  

*R*[*F* ^2^ > 2σ(*F* ^2^)] = 0.066
*wR*(*F* ^2^) = 0.208
*S* = 1.04
3454 reflections
145 parameters
1 restraint
H-atom parameters constrained
Δρ_max_ = 0.28 e Å^−3^
Δρ_min_ = −0.22 e Å^−3^

### 

Data collection: *APEX2* (Bruker, 2004 ▸); cell refinement: *APEX2* and *SAINT* (Bruker, 2004 ▸); data reduction: *SAINT* and *XPREP* (Bruker, 2004 ▸); program(s) used to solve structure: *SHELXS97* (Sheldrick, 2008 ▸); program(s) used to refine structure: *SHELXL97* (Sheldrick, 2008 ▸); molecular graphics: *ORTEP-3 for Windows* (Farrugia, 2012 ▸); software used to prepare material for publication: *SHELXL97*.

## Supplementary Material

Crystal structure: contains datablock(s) I, New_Global_Publ_Block. DOI: 10.1107/S2056989015018307/zs2344sup1.cif

Structure factors: contains datablock(s) I. DOI: 10.1107/S2056989015018307/zs2344Isup2.hkl

Supporting information file. DOI: 10.1107/S2056989015018307/zs2344Isup3.cml

**Fig. 1.**
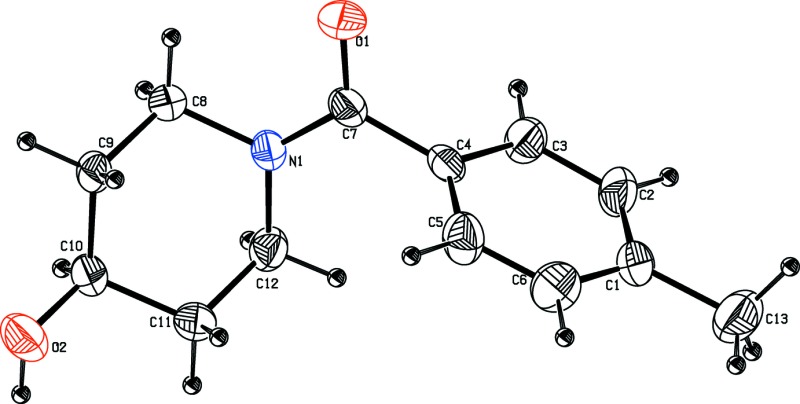
The molecular structure and atom numbering scheme for the title compound, with displacement ellipsoids drawn at the 30% probability level.

**Fig. 2.**
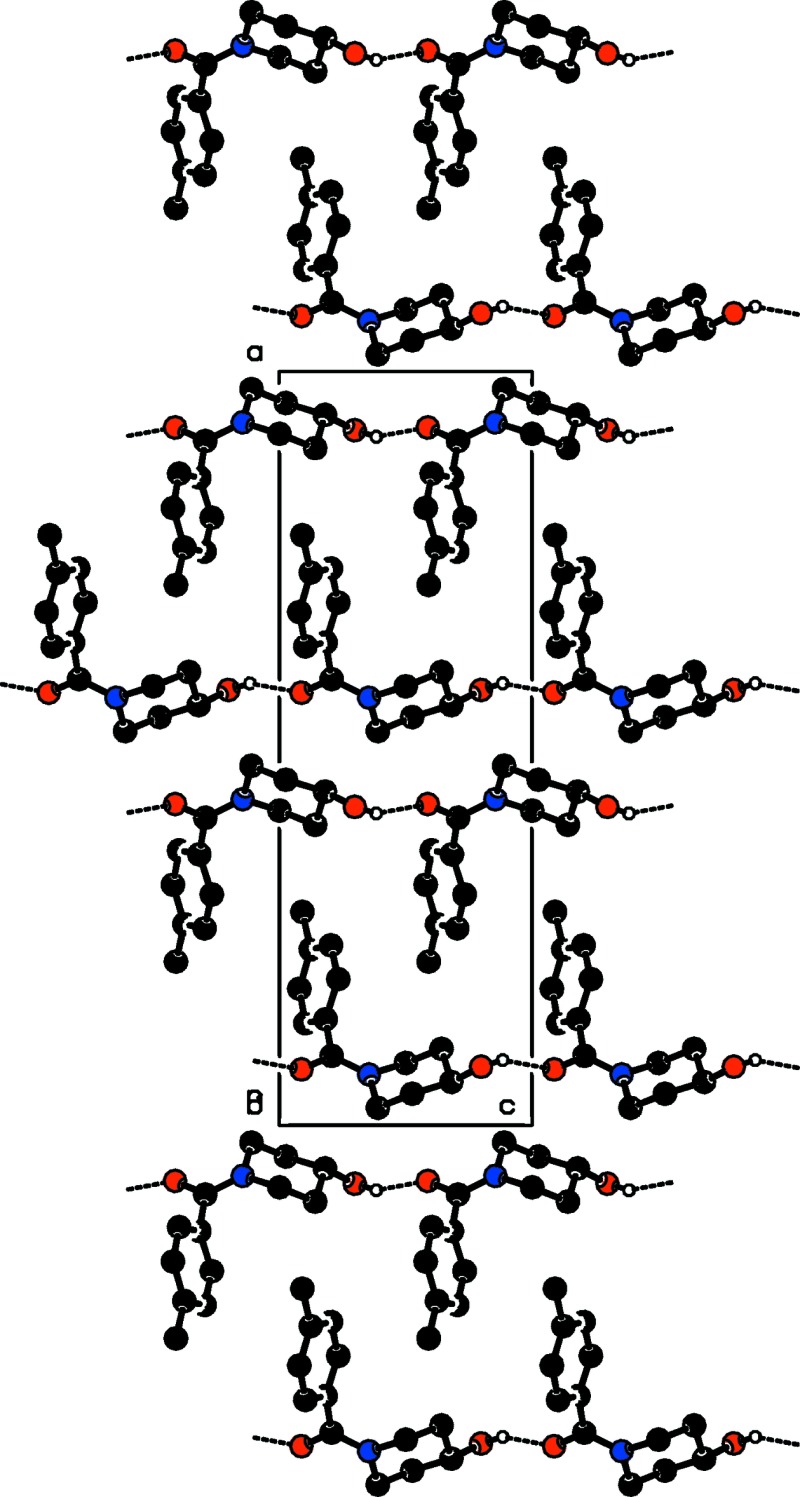
The crystal packing in the unit cell viewed along b. The dashed lines indicate hydrogen bonds.

. DOI: 10.1107/S2056989015018307/zs2344fig1.tif
The mol­ecular structure and atom numbering scheme for the title compound, with displacement ellipsoids drawn at the 30% probability level.

b . DOI: 10.1107/S2056989015018307/zs2344fig2.tif
The crystal packing in the unit cell viewed along *b*. The dashed lines indicate hydrogen bonds.

CCDC reference: 1428660

Additional supporting information:  crystallographic information; 3D view; checkCIF report

## Figures and Tables

**Table 1:** Hydrogen-bond geometry (, )

*D*H*A*	*D*H	H*A*	*D* *A*	*D*H*A*
O2H2*A*O1^i^	0.82	1.97	2.741(4)	156

Symmetry code: (i) 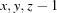.

## supplementary crystallographic information

### S1. Comment

The piperidine ring is one of the most recognizable structural entities among heterocyclic molecules (Katritzky, 1995). A piperidine series of gamma-secretase inhibitors have been evaluated for treatment of Alzheimer's disease (AD) (Pissamitski *et al.*, 2007). Some piperidines were found to possess high profile biological activities, including cytotoxic and anticancer properties (Dimmock *et al.*, 2001). The piperidine ring is a feature of oral anaesthetics and narcotic analgesics (Watson *et al.*, 2000); Thomas *et al.*, 1998). Piperidine derivatives are used clinically to prevent post-operative vomiting, to speed up gastric emptying before anaesthesia, to facilitate radiological investigations and to correct a variety of disturbances of gastrointestinal functions (Sambath *et al.*, 2004).

The title compound, C_13_H_17_NO_2_, has been synthesized and the structure (Fig. 1) is reported herein. In this compound, The C—C distances in the piperidine ring and the benzene ring are in the range 1.497 (6)–1.515 (5) Å and 1.357 (6)–1.386 (5) Å, respectively and are comparable with literature values. The C—N distances in the piperidine ring are 1.455 (5) Å and 1.462 (5) Å] and are in good agreement with values in a similar reported structure (Revathi *et al.*, 2015). The C7—O1 distance is 1.238 (5) Å, indicating double bond character and is comparable with the value reported previously (Prathebha *et al.*, 2015). The dihedral angle between piperidine and benzene rings is 51.7 (2)°. The bond angle sum around the N1 atom are 359.8 (3)° indicating an *sp*^2^ hybridization of the atoms. The C8—N1—C7—O1 torsion angle [-9.0 (6)°] indicates that the keto group is in a *syn-periplanar* (-*sp*) orientation with respect to the piperidine ring which adopts a chair conformation, with puckering parameters of q2 = 0.016 (4) Å, φ2 = 168.41° q3 = -0.560 (4) Å, QT = 0.561 (4) Å and θ2 = 178.38 (4)°.

The crystal packing is stabilized by a single intermolecular O2—H···O1^i^ hydrogen bond (Table 1), forming one-dimensional chains which extend along [001] (Fig. 2). Present also in the structure is a short intramolecular C8—H···O1 interaction [2.740 (5) Å].

### S2. Experimental

The title compound was synthesized using a published procedure (Revathi *et al.*, 2015). In a 250 ml round-bottomed flask, 120 mL of ethyl methyl ketone was added to 4-hydroxypiperdine (0.02 mol) and stirred at room temperature. After 5 min, triethylamine (0.04 mol) was added and the mixture was stirred for 15 min. 4-Methyl benzoyl chloride (0.04 mol) was then added and the reaction mixture was stirred at room temperature for *ca.* 2 h. A white precipitate of triethylammonium chloride was formed, which was removed by filtration and the filtrate was evaporated to give the crude product. This was recrystallized twice from ethyl methyl ketone giving colourless block-like crystals of the title compound (yield: 82%).

### S3. Refinement

H atoms were positioned geometrically and treated as riding on their parent atoms and refined with C—H distances of 0.93–0.98 Å and an O—H distance of 0.82 Å, with *U*_iso_(H) = 1.5 *U*_eq_(C-methyl and O), *U*_iso_(H)= 1.2*U*eq(C, O) for other H atoms. One reflection (2 0 0) was considered to be affected by the beamstop and was omitted. The value of the absolute structure factor (Flack, 1983), although not of particular relevance but meaningless in this structure, was determined as 0(3) for 1610 Friedel pairs.

### Crystal data

**Table:**

C_13_H_17_NO_2_	*F*(000) = 472
*M**_r_* = 219.28	*D*_x_ = 1.197 Mg m^−^^3^
Orthorhombic, *P**c**a*2_1_	Mo *K*α radiation, λ = 0.71073 Å
Hall symbol: P 2c -2ac	θ = 1.7–29.8°
*a* = 23.933 (5) Å	µ = 0.08 mm^−^^1^
*b* = 6.3317 (12) Å	*T* = 293 K
*c* = 8.0269 (14) Å	Block, colourless
*V* = 1216.3 (4) Å^3^	0.24 × 0.22 × 0.22 mm
*Z* = 4	

### Data collection

**Table:**

Bruker Kappa APEXII CCD diffractometer	3454 independent reflections
Radiation source: fine-focus sealed tube	1668 reflections with *I* > 2σ(*I*)
Graphite monochromator	*R*_int_ = 0.039
ω and φ scans	θ_max_ = 29.8°, θ_min_ = 3.1°
Absorption correction: multi-scan (*SADABS*; Bruker, 2004)	*h* = −33→30
*T*_min_ = 0.981, *T*_max_ = 0.985	*k* = −8→8
10595 measured reflections	*l* = −10→11

### Refinement

**Table:**

Refinement on *F*^2^	Primary atom site location: structure-invariant direct methods
Least-squares matrix: full	Secondary atom site location: difference Fourier map
*R*[*F*^2^ > 2σ(*F*^2^)] = 0.066	Hydrogen site location: inferred from neighbouring sites
*wR*(*F*^2^) = 0.208	H-atom parameters constrained
*S* = 1.04	*w* = 1/[σ^2^(*F*_o_^2^) + (0.121*P*)^2^ + 0.297*P*] where *P* = (*F*_o_^2^ + 2*F*_c_^2^)/3
3454 reflections	(Δ/σ)_max_ < 0.001
145 parameters	Δρ_max_ = 0.28 e Å^−^^3^
1 restraint	Δρ_min_ = −0.22 e Å^−^^3^

### Special details

**Table:**

Geometry. All esds (except the esd in the dihedral angle between two l.s. planes) are estimated using the full covariance matrix. The cell esds are taken into account individually in the estimation of esds in distances, angles and torsion angles; correlations between esds in cell parameters are only used when they are defined by crystal symmetry. An approximate (isotropic) treatment of cell esds is used for estimating esds involving l.s. planes.
Refinement. Refinement of F^2^ against ALL reflections. The weighted R-factor wR and goodness of fit S are based on F^2^, conventional R-factors R are based on F, with F set to zero for negative F^2^. The threshold expression of F^2^ > 2sigma(F^2^) is used only for calculating R-factors(gt) etc. and is not relevant to the choice of reflections for refinement. R-factors based on F^2^ are statistically about twice as large as those based on F, and R- factors based on ALL data will be even larger.

### Fractional atomic coordinates and isotropic or equivalent isotropic displacement parameters (Å^2^)

**Table:**

	*x*	*y*	*z*	*U*_iso_*/*U*_eq_	
O2	0.42250 (13)	0.9019 (5)	0.2011 (4)	0.0766 (9)	
H2A	0.4137	0.8390	0.1158	0.115*	
N1	0.43269 (12)	0.5768 (5)	0.6446 (4)	0.0502 (8)	
O1	0.42609 (13)	0.6719 (6)	0.9120 (3)	0.0885 (11)	
C10	0.44267 (16)	0.7574 (6)	0.3187 (5)	0.0533 (9)	
H10	0.4759	0.6870	0.2737	0.064*	
C4	0.35949 (13)	0.4254 (6)	0.8163 (4)	0.0482 (8)	
C1	0.26666 (15)	0.1641 (8)	0.8753 (5)	0.0628 (11)	
C12	0.41754 (16)	0.4528 (6)	0.4989 (5)	0.0545 (9)	
H12A	0.3877	0.3558	0.5279	0.065*	
H12B	0.4495	0.3701	0.4632	0.065*	
C7	0.40802 (15)	0.5691 (6)	0.7925 (4)	0.0505 (9)	
C11	0.39864 (16)	0.5930 (6)	0.3578 (4)	0.0524 (9)	
H11A	0.3640	0.6627	0.3883	0.063*	
H11B	0.3916	0.5077	0.2596	0.063*	
C6	0.26126 (15)	0.3547 (9)	0.7954 (6)	0.0758 (13)	
H6	0.2261	0.3976	0.7599	0.091*	
C5	0.30623 (16)	0.4839 (7)	0.7665 (6)	0.0669 (11)	
H5	0.3009	0.6125	0.7128	0.080*	
C2	0.31909 (18)	0.1055 (7)	0.9206 (6)	0.0742 (13)	
H2	0.3245	−0.0261	0.9694	0.089*	
C8	0.47699 (16)	0.7294 (7)	0.6096 (5)	0.0598 (10)	
H8A	0.5107	0.6557	0.5758	0.072*	
H8B	0.4853	0.8099	0.7094	0.072*	
C3	0.36470 (16)	0.2363 (7)	0.8960 (6)	0.0679 (12)	
H3	0.3996	0.1943	0.9346	0.082*	
C9	0.45847 (17)	0.8752 (5)	0.4737 (5)	0.0527 (9)	
H9A	0.4884	0.9732	0.4482	0.063*	
H9B	0.4266	0.9566	0.5121	0.063*	
C13	0.2170 (2)	0.0223 (10)	0.9085 (8)	0.0951 (16)	
H13A	0.1836	0.0885	0.8674	0.143*	
H13B	0.2135	−0.0011	1.0262	0.143*	
H13C	0.2223	−0.1105	0.8530	0.143*	

### Atomic displacement parameters (Å^2^)

**Table:**

	*U*^11^	*U*^22^	*U*^33^	*U*^12^	*U*^13^	*U*^23^
O2	0.097 (2)	0.0792 (19)	0.0541 (17)	0.0001 (17)	−0.0073 (14)	0.0175 (16)
N1	0.0614 (17)	0.0478 (18)	0.0413 (15)	−0.0129 (14)	0.0013 (12)	0.0037 (14)
O1	0.105 (2)	0.117 (3)	0.0441 (16)	−0.045 (2)	−0.0064 (15)	−0.0106 (18)
C10	0.0621 (19)	0.056 (2)	0.0418 (18)	−0.0096 (18)	0.0032 (15)	0.0054 (18)
C4	0.056 (2)	0.056 (2)	0.0323 (15)	0.0001 (16)	−0.0009 (13)	0.0075 (17)
C1	0.062 (2)	0.079 (3)	0.0472 (19)	−0.016 (2)	0.0102 (17)	0.005 (2)
C12	0.061 (2)	0.046 (2)	0.056 (2)	−0.0113 (17)	0.0065 (16)	−0.002 (2)
C7	0.0619 (19)	0.050 (2)	0.0396 (18)	−0.0035 (16)	−0.0048 (16)	0.0088 (17)
C11	0.061 (2)	0.055 (2)	0.0422 (17)	−0.0058 (17)	−0.0002 (14)	−0.0092 (18)
C6	0.0438 (19)	0.100 (3)	0.084 (3)	0.006 (2)	0.003 (2)	0.005 (3)
C5	0.064 (2)	0.060 (2)	0.077 (3)	0.0036 (19)	0.003 (2)	0.021 (2)
C2	0.075 (3)	0.064 (3)	0.083 (3)	−0.020 (2)	−0.012 (2)	0.032 (3)
C8	0.064 (2)	0.067 (3)	0.048 (2)	−0.0260 (19)	−0.0126 (16)	0.010 (2)
C3	0.059 (2)	0.061 (3)	0.084 (3)	−0.0081 (19)	−0.019 (2)	0.028 (2)
C9	0.076 (2)	0.0356 (18)	0.0470 (18)	−0.0178 (18)	−0.0010 (16)	0.0040 (18)
C13	0.078 (3)	0.121 (4)	0.087 (3)	−0.041 (3)	0.016 (3)	−0.019 (3)

### Geometric parameters (Å, º)

**Table:**

O2—C10	1.401 (5)	C12—H12B	0.9700
O2—H2A	0.8200	C11—H11A	0.9700
N1—C7	1.327 (5)	C11—H11B	0.9700
N1—C12	1.454 (5)	C6—C5	1.372 (6)
N1—C8	1.462 (4)	C6—H6	0.9300
O1—C7	1.237 (5)	C5—H5	0.9300
C10—C9	1.500 (5)	C2—C3	1.385 (5)
C10—C11	1.514 (5)	C2—H2	0.9300
C10—H10	0.9800	C8—C9	1.496 (5)
C4—C3	1.363 (6)	C8—H8A	0.9700
C4—C5	1.386 (5)	C8—H8B	0.9700
C4—C7	1.487 (5)	C3—H3	0.9300
C1—C2	1.358 (6)	C9—H9A	0.9700
C1—C6	1.373 (7)	C9—H9B	0.9700
C1—C13	1.514 (6)	C13—H13A	0.9600
C12—C11	1.509 (5)	C13—H13B	0.9600
C12—H12A	0.9700	C13—H13C	0.9600
			
C10—O2—H2A	109.5	C5—C6—C1	122.0 (4)
C7—N1—C12	126.1 (3)	C5—C6—H6	119.0
C7—N1—C8	121.3 (3)	C1—C6—H6	119.0
C12—N1—C8	112.6 (3)	C6—C5—C4	120.9 (4)
O2—C10—C9	108.7 (3)	C6—C5—H5	119.6
O2—C10—C11	110.4 (3)	C4—C5—H5	119.6
C9—C10—C11	110.2 (3)	C1—C2—C3	121.8 (4)
O2—C10—H10	109.1	C1—C2—H2	119.1
C9—C10—H10	109.1	C3—C2—H2	119.1
C11—C10—H10	109.1	N1—C8—C9	109.4 (3)
C3—C4—C5	117.0 (3)	N1—C8—H8A	109.8
C3—C4—C7	121.7 (3)	C9—C8—H8A	109.8
C5—C4—C7	121.2 (3)	N1—C8—H8B	109.8
C2—C1—C6	116.9 (3)	C9—C8—H8B	109.8
C2—C1—C13	121.1 (4)	H8A—C8—H8B	108.2
C6—C1—C13	122.0 (4)	C4—C3—C2	121.3 (4)
N1—C12—C11	111.1 (3)	C4—C3—H3	119.3
N1—C12—H12A	109.4	C2—C3—H3	119.3
C11—C12—H12A	109.4	C8—C9—C10	111.9 (3)
N1—C12—H12B	109.4	C8—C9—H9A	109.2
C11—C12—H12B	109.4	C10—C9—H9A	109.2
H12A—C12—H12B	108.0	C8—C9—H9B	109.2
O1—C7—N1	121.2 (3)	C10—C9—H9B	109.2
O1—C7—C4	119.7 (3)	H9A—C9—H9B	107.9
N1—C7—C4	119.0 (3)	C1—C13—H13A	109.5
C12—C11—C10	110.6 (3)	C1—C13—H13B	109.5
C12—C11—H11A	109.5	H13A—C13—H13B	109.5
C10—C11—H11A	109.5	C1—C13—H13C	109.5
C12—C11—H11B	109.5	H13A—C13—H13C	109.5
C10—C11—H11B	109.5	H13B—C13—H13C	109.5
H11A—C11—H11B	108.1		
			
C7—N1—C12—C11	117.5 (4)	C13—C1—C6—C5	179.5 (5)
C8—N1—C12—C11	−58.2 (4)	C1—C6—C5—C4	0.5 (7)
C12—N1—C7—O1	175.6 (4)	C3—C4—C5—C6	−0.6 (6)
C8—N1—C7—O1	−9.0 (6)	C7—C4—C5—C6	−177.1 (4)
C12—N1—C7—C4	−1.3 (6)	C6—C1—C2—C3	3.5 (7)
C8—N1—C7—C4	174.1 (3)	C13—C1—C2—C3	−177.9 (5)
C3—C4—C7—O1	−74.8 (5)	C7—N1—C8—C9	−117.4 (4)
C5—C4—C7—O1	101.5 (5)	C12—N1—C8—C9	58.6 (4)
C3—C4—C7—N1	102.2 (4)	C5—C4—C3—C2	2.2 (7)
C5—C4—C7—N1	−81.6 (5)	C7—C4—C3—C2	178.7 (4)
N1—C12—C11—C10	54.7 (4)	C1—C2—C3—C4	−3.8 (8)
O2—C10—C11—C12	−173.3 (3)	N1—C8—C9—C10	−57.0 (4)
C9—C10—C11—C12	−53.1 (4)	O2—C10—C9—C8	176.3 (3)
C2—C1—C6—C5	−1.9 (7)	C11—C10—C9—C8	55.1 (5)

### Hydrogen-bond geometry (Å, º)

**Table:**

*D*—H···*A*	*D*—H	H···*A*	*D*···*A*	*D*—H···*A*
O2—H2*A*···O1^i^	0.82	1.97	2.741 (4)	156
C8—H8*B*···O1	0.97	2.33	2.740 (5)	105

Symmetry code: (i) *x*, *y*, *z*−1.
