# Supplementary figures and images for: Crystal structure of (4-hy­droxy­piperidin-1-yl)(4-methyl­phen­yl)methanone
Source: Acta Crystallogr E Crystallogr Commun. 2015 Oct 3;71(Pt 11):o817–8. doi: 10.1107/S2056989015018307 (PMC4645002; doi:10.1107/S2056989015018307)

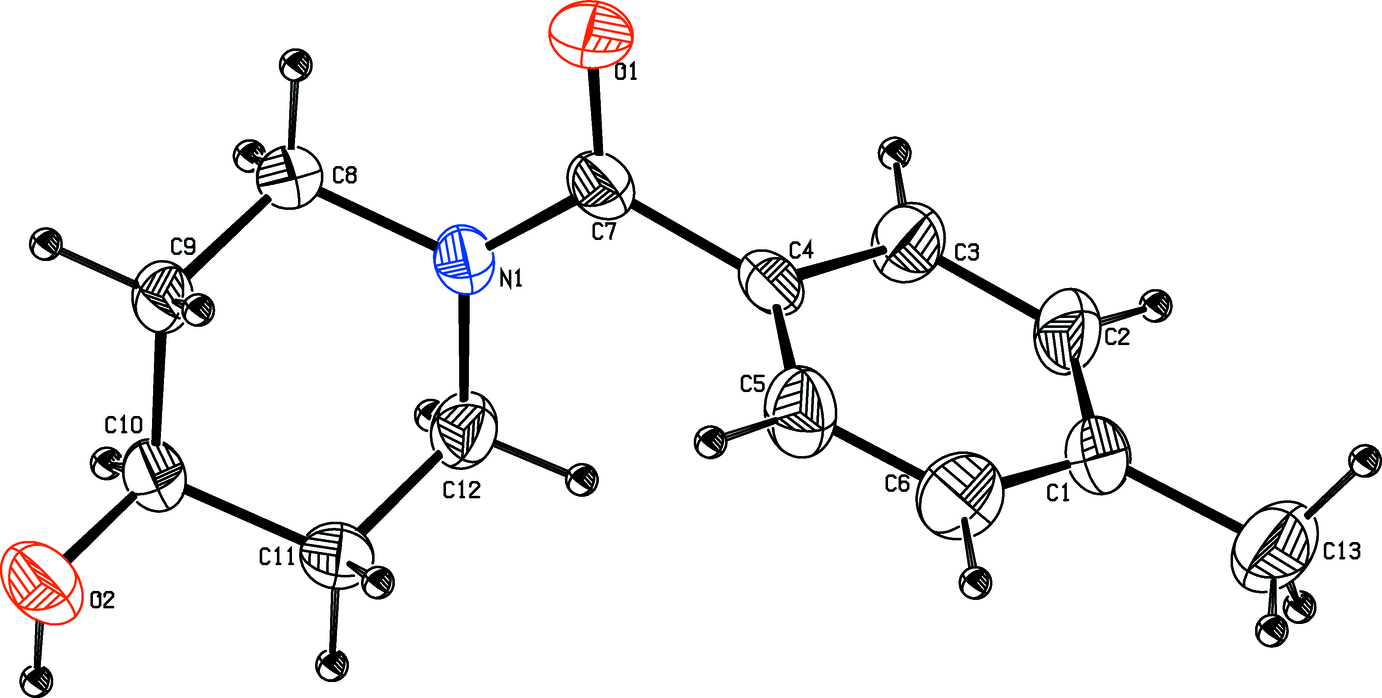

Supplement: Supplementary file 4 [file e-71-0o817-fig1.tif]

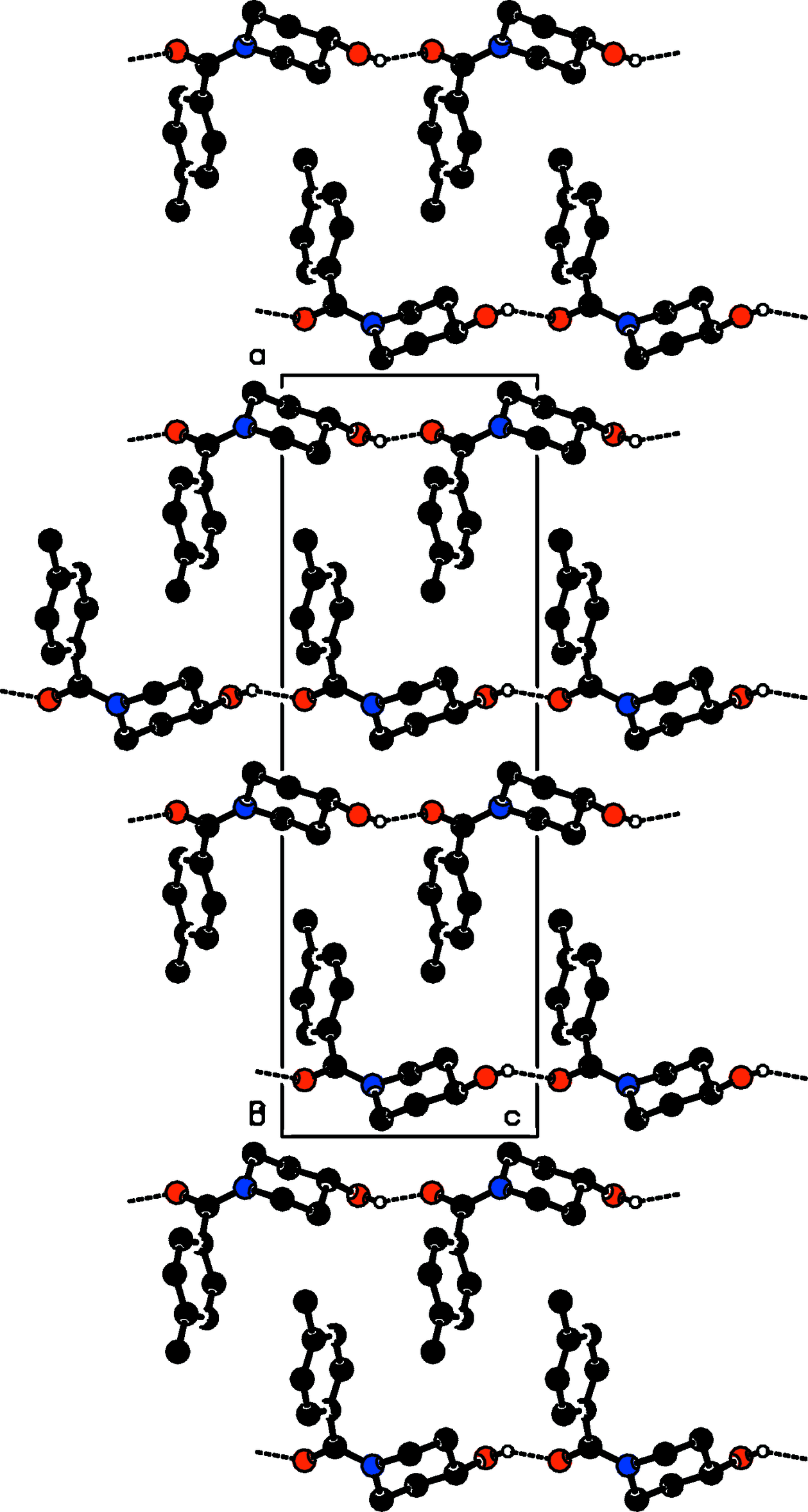

Supplement: Supplementary file 5 [file e-71-0o817-fig2.tif]
